# International Expert Consensus on Instrument-Assisted Soft-Tissue Mobilization Precautions and Contraindications: A Modified Delphi Study

**DOI:** 10.3390/healthcare13060642

**Published:** 2025-03-15

**Authors:** Scott W. Cheatham, Russell T. Baker, M. Terry Loghmani, Robert Schleip

**Affiliations:** 1 Department of Kinesiology, California State University Dominguez Hills, Carson, CA 90747, USA; 2 Department of Movement Sciences, University of Idaho, Moscow, ID 83844, USA; 3 WWAMI Medical Education Program, University of Idaho, Moscow, ID 83844, USA; russellb@uidaho.edu; 4 Idaho Office of Underserved and Rural Medical Research, University of Idaho, Moscow, ID 83844, USA; 5 Department of Physical Therapy, Indiana University, Indianapolis, IN 46202, USA; mloghman@iu.edu; 6 Conservative and Rehabilitative Orthopedics, TUM School of Medicine and Health, Technical University of Munich, 80333 Munich, Germany; robert.schleip@tum.de; 7 Department for Medical Professions, Diploma Hochschule, 37242 Bad Sooden Allendorf, Germany

**Keywords:** Graston^®^, myofascial, fascia, massage, treatment

## Abstract

**Background**: Instrument-assisted soft-tissue mobilization (IASTM) is a popular myofascial intervention used by healthcare professionals. Despite the growing body of research evidence, there is still a gap in understanding what healthcare professionals consider as treatment precautions or contraindications. To date, no consensus on precautions and contraindications has been established among IASTM experts. The purpose of this modified Delphi survey was to determine IASTM precautions and contraindications among international IASTM experts. **Methods**: A three-round Delphi study of 24 international IASTM experts was conducted. In round 1, experts chose from a list of 81 medical conditions and treatment considerations that could be a concern for IASTM treatment. Consensus was considered if more than 70% of experts agreed on an item. Round 2 included the updated list of 39 items, and the experts decided if each item should be a precaution, contraindication, or both. The *strength of agreement grade scale* was used to rank the precautions and contraindications, by the level of expert agreement using grades A-D (e.g., A-strong, B-moderate, C-weak, D-both). Grade D conditions could potentially be both a precaution and contraindication. In round 3, the final list of categories and items was presented to the experts for final approval. Results: All recruited experts (*n* = 24) participated in the three rounds and the final list of items received 100% approval. Contraindications had the highest number of conditions (*n* = 16) across the strength of agreement grade categories A–C followed by category D (both) (*n* = 8). **Discussion**: This Delphi study was the first survey to document expert consensus on precautions and contraindications based upon the strength of agreement. This study offers a beginner’s guide for clinicians to safely implement IASTM by establishing required precautions and contraindications through consensus agreement. **Conclusions**: This survey should be the first step in a series of planned IASTM studies on precautions and contraindications to establish the best-practice recommendations for the application of IASTM in clinical practice.

## 1. Introduction

Instrument-assisted soft-tissue mobilization (IASTM) is a popular myofascial intervention used by healthcare professionals in the treatment of different musculoskeletal conditions. IASTM treatment is based upon the rationale by James Cyriax [1] with the first study published in 1977 [2]. Since then, IASTM has become a commonly researched intervention with demonstrated efficacy for the treatment of different musculoskeletal conditions [3,4,5].

Despite the growing body of research evidence, there is still a gap in understanding what precautions and contraindications are considered by healthcare professionals for IASTM treatment [1]. Among the current published literature, only one published narrative review was identified with proposed specific precautions and contraindications for IASTM treatment [1]. Most of the guidance for IASTM treatment precautions and contraindications has come from IASTM manufacturers through their continuing education courses [6,7,8,9] and a 2008 case series that details relative and absolute contraindications based upon the Graston Technique^®^ continuing education course [9,10]. To date, no consensus on IASTM treatment precautions and contraindications has been established among IASTM experts.

Determining potential precautions and contraindications prior to IASTM treatment is foundational for safe patient treatment. Overlooking or not considering potentially unsafe medical conditions prior to IASTM treatment may increase the risk for negative patient consequences or legal implications. Given the lack of empirical evidence to guide IASTM application for treatment precautions and contraindications, expert consensus to determine qualifying conditions would be beneficial to guide clinical practice. The development of expert consensus would help establish precaution and contraindication recommendations for healthcare professionals who treat diverse patient populations with IASTM. Thus, the purpose of this study was to use a modified Delphi survey approach to establish agreed-upon IASTM precautions and contraindications among international IASTM experts.

## 2. Materials and Methods

### 2.1. Study Design

A modified 3-round Delphi study design was implemented to establish a consensus on IASTM precautions and contraindications among IASTM experts. The methodological approach was based upon the methodology of prior similar myofascial Delphi surveys [11,12]. The Delphi process involves achieving a consensus of opinions among experts on a specific topic over a series of rounds [12]. This study was approved by California State University Dominguez Hill’s Institutional Review Board (IRB# 2025-26).

### 2.2. Expert Recruitment

IASTM experts were recruited to participate in this study. Inclusion criteria included being a healthcare professional and one or more of the following criteria: current or prior teaching of IASTM to students or healthcare professionals, actively researching IASTM and other myofascial interventions, use of IASTM within their patient treatments, or being an author on one or more peer-reviewed IASTM publications. Exclusion criteria included professionals not meeting the inclusions or being non-responsive to the survey email invitation. The a priori goal was to have a minimum of 20 contributing experts for each round of the Delphi process [13,14]. IASTM experts were identified through a comprehensive search of IASTM and myofascial social media interest groups, peer-reviewed publications, and professional continuing education organizations. These methods have been used in prior related Delphi survey studies [12]. Nominated experts received an email invitation to participate in the study. Study participants remained anonymous during all rounds of the survey. Participants were contacted only to be notified of each round of the survey with relevant information [11,12].

### 2.3. Survey Development and Rounds

The survey was initially developed by the lead author (S.W.C.) and reviewed by the IASTM modified Delphi survey committee. The committee consisted of three healthcare IASTM experts who were a doctorate-level physical therapist, doctorate-level certified athletic trainer, and doctorate-level manual therapist. All committee experts had researched IASTM, with peer-reviewed publications and over 15 years of professional experience with IASTM and other myofascial interventions. The survey underwent two rounds of expert committee review, feedback, and development, per the methodology of prior related Delphi survey studies [11,12].

The survey was derived from a systematic search of the research evidence on IASTM precautions and contraindications between March 1997 and September 2024. The PubMed, Google Scholar, OneSearch, and CINAHL databases were searched using the following terms individually or in combination: augmented soft-tissue mobilization, instrument-assisted soft-tissue mobilization or IASTM, instrument-assisted soft-tissue treatment, instrument-assisted cross-fiber massage, instrument-assisted neuromobilization, ASTYM^®^, Graston^®^ treatment, humans, precautions, and contraindications [1,12]. A direct search of IASTM continuing education company education materials was also conducted [6,7,8]. Precautions were defined as a medical condition that could increase the risk of an adverse reaction and needs to be monitored by the healthcare professional, while a contraindication was a medical condition that would be deemed unsafe for IASTM treatment [1].

The final survey included initial questions related to participant demographics such as self-identified gender, chronological age, years of clinical experience, nationality, healthcare specialty, primary work setting, and years of clinical experience using IASTM. The survey also included 81 identified medical conditions or treatment considerations for expert consideration as a precaution, contraindication, or both allocated under the following categories: (1) musculoskeletal conditions; (2) cardiovascular, respiratory, and chronic conditions; (3) integumentary, connective tissue, nervous system, and psychological conditions; and (4) miscellaneous conditions and treatment considerations (Table 1) (see Supplementary Materials for glossary of terms). These items were adapted from a prior IASTM narrative review [1], a 2008 case series [10], IASTM manufacturer continuing education courses [6,7,8], and the systematic review of the literature. Each medical condition category had specific questions in the 3-round survey, which will be detailed below [11,12].

The 3-round survey was developed using the Qualtrics online platform (Qualtrics, 333 W. River Park Drive Provo, UT 84604 USA). Experts accessed each survey (rounds 1 to 3) via a survey link sent through an email. Each link corresponded to the specific Delphi round.

#### 2.3.1. Survey Round 1

The initial survey included participant demographic questions, and specific questions related to the 81 identified medical conditions or treatment considerations allocated into specific categories. Each category question included the medical conditions or treatment considerations relevant to IASTM (Table 1). Participants ranked their agreement for each condition or treatment consideration using a 6-point Likert scale (1 = strongly disagree; 6 = strongly agree), which has been used in prior related Delphi surveys [11]. Participants also had an opportunity to provide comments in an open text field question to allow for consideration of other conditions or considerations not mentioned in the item lists presented in Table 1 [11]. The goal of the round 1 survey was to identify potential conditions and treatment considerations that could be either a precaution or contraindication or both for IASTM that would be considered by the expert panel in round 2. Question item consensus was considered to be ≥70% agreement among IASTM survey participants [11,15]. The IASTM Delphi survey committee reviewed all participant responses within the medical condition categories (e.g., musculoskeletal conditions) from the round 1 online survey. Those question items that met or exceeded the expert consensus threshold (≥70%) were then extracted and placed into the round 2 survey within their respective categories for experts to review.

#### 2.3.2. Survey Round 2

The second survey included the updated list of 39 medical conditions and treatment considerations based upon the ≥ 70% agreement among IASTM experts in round 1. Round 2 also included similar category question items as the prior round. For each question, expert participants determined if each qualifying condition or treatment consideration (from round 1) should be a precaution, contraindication, or both. Two additional questions related to the round 1 open comments were included to capture and qualify other medical conditions not mentioned in the original list. Once the survey participants completed round 2, the IASTM Delphi survey committee reviewed all responses. The committee then extracted and compiled the final list of 39 items and placed them within their respective medical condition categories. Survey answers were further stratified as precautions, contraindications, or both, based upon the strength of expert agreement.

The *strength of agreement grade scale* (grade A-D) was created to help rank the strength of consensus among experts for conditions or considerations that were classified as precautions, contraindications, or both in round 2 (Table 2). This scale also demonstrates the diversity that occurs in clinical practice and that some professionals may not agree that specific conditions are absolute precautions or contraindications. Healthcare professionals may have different treatment approaches, philosophies, and beliefs based upon the IASTM research evidence [11]. Question items that did not meet or exceed the ≥50% expert agreement threshold for a single classification (i.e., greater than 50% agreement that the condition should be considered as a precaution or contraindication) were classified as grade D (both) (i.e., a potential precaution and contraindication). It is recommended that items in grade D be considered at minimum an IASTM potential precaution by healthcare professionals. For survey round 3, the final list was presented to the survey participants for final approval.

#### 2.3.3. Survey Round 3

**Figure 1:**
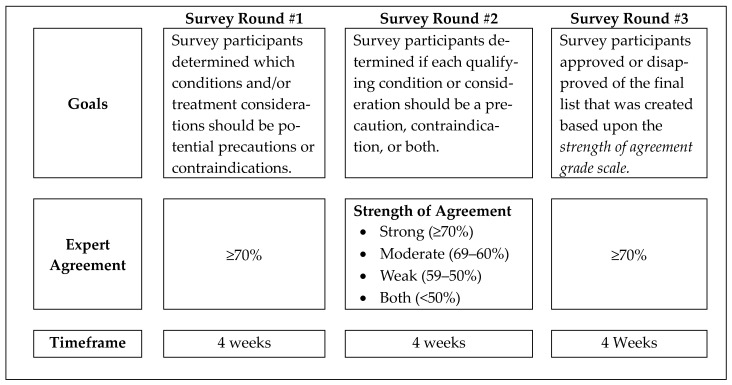
Overview of the 3-round Delphi survey process.

This last round included a list of 39 final medical conditions and treatment considerations classified as precautions, contraindications, or both using the *strength of agreement grade scale* from round 2. The IASTM Delphi survey committee compiled and presented the final list (within their respective medical condition category) to expert participants. The experts then reviewed the final list of classified items and documented their approval or disapproval. Expert consensus for the final list was considered to be ≥70% agreement among participants [11,15]. A summary of the three-round Delphi survey process is shown in Figure 1 [11].

#### 2.3.4. Data Processing

Anonymous survey responses were collected using an electronic survey questionnaire (Qualtrics, 333 W. River Park Drive Provo, UT 84604 USA) between September 2024 and January 2025. Data were downloaded from the questionnaire platform and descriptive data analysis was performed to evaluate the responses from the Likert scale using Microsoft Excel^®^ (Redmond, WA, USA). Open text comments were compiled and processed by the IASTM Delphi survey committee [11].

## 3. Results

A total of 24 IASTM experts (men = 18, women = 6; mean age = 52 years ± 8.25; range = 32–65 years) consented to participate in the three-round survey which included round #1 demographic questions. IASTM expert participants consisted of physical therapists (n = 13), chiropractors (n = 6), licensed/certified athletic trainers (4), and an occupational therapist (n = 1). The experts represented countries including the United States (n = 16), Canada (n = 1), Greece (N = 4), United Kingdom (n = 1), Belgium (n = 1), and no response (n = 1). The majority of experts reported working in a private outpatient healthcare facility (52%) followed by an academic/research institution (19%), sports performance facility (10%), and other settings (19%). IASTM experts reported practicing an average of 26 years ± 9.5 (range 6 to 44 years) and using IASTM for an average of 15 years ± 5.25 (range 7 to 28 years) (see Supplementary Materials on IASTM expert panel).

In survey round 1, we received 24/24 (100%) completed survey responses. This round included 81 identified medical conditions or treatment considerations allocated under their respective categories. The expert participants chose a total of 39 conditions or treatment considerations that were moved to round 2 of the survey. In survey round 2, we received 24/24 (100%) completed responses for all question items except for three conditions that received 23/24 (96%). Survey participants decided if the 39 conditions or treatment considerations should be classified as precautions, contraindications, or both. Upon round 2 completion, the IASTM survey committee compiled the final list of precautions, contraindications, or both using the strength of agreement grading scale. The final list was moved on to the next round. In survey round 3, we received 24/24 responses and 100% agreement among IASTM experts for the final list of precautions, contraindications, or both as classified using the strength of agreement scale (Table 3, Table 4 and Table 5).

## 4. Discussion

Healthcare professionals use IASTM to treat numerous musculoskeletal conditions; however, little is known about what precautions and contraindications should be considered by these professionals when applying IASTM [1,4]. Determining potential precautions and contraindications prior to IASTM treatment is foundational for safe patient treatment. Given the lack of empirical evidence to guide IASTM application for treatment precautions and contraindications, expert consensus to determine qualifying conditions would be beneficial to guide clinical practice. Development of expert consensus would help establish precaution and contraindication recommendations for healthcare professionals who treat diverse patient populations with IASTM. Thus, the purpose of this study was to use a modified Delphi survey approach to establish agreed-upon IASTM precautions and contraindications among international IASTM experts.

The IASTM expert agreement of the final set of 39 medical conditions and treatment considerations was high (100% agreement) and provides initial guidance for healthcare professionals who use IASTM. This study was unique due to the further classification of conditions using the *strength of agreement grade scale*, which allowed the classification of medical conditions and treatment considerations as strong, moderate, weak, or both for precautions and contraindications based upon expert consensus.

In clinical practice, medical conditions and clinical considerations may not always be absolute precautions or contraindications for patients. A precautionary medical condition for one patient may be a contraindication for another patient, and a consideration (e.g., open wound, suture site, etc.) may be a precaution or contraindication for IASTM application depending on where or how IASTM application is occurring for a given patient. Healthcare professionals may also consider IASTM precautions and contraindications differently than their peers, which represents diversity in clinical practice. The use of Delphi or modified Delphi procedures around IASTM application allow for clinical recommendations based upon expert consensus that may further guide clinical decision making when other established evidence is lacking [12,13,14,15,16].

Healthcare professionals should consider the final list of items are a starting point and not all-inclusive. Other medical conditions or treatment considerations may exist beyond this study that may be considered IASTM precautions or contraindications or both for specific patients [1]. A medical condition or treatment consideration in the grade D category (both)should at minimum be considered a precaution by the healthcare professional. For example, healing surgical scars may be both a precaution and/or contraindication for some patients based upon the stage of healing, or if IASTM application is being applied to a patient somewhere else on the body. At minimum, the healthcare professional should consider these situations as precautions and closely monitor the patient’s response during and after an IASTM treatment. Awareness and consideration of the different conditions and patient scenarios will ensure minimal consideration to ensure patient safety prior to and during IASTM treatment. Healthcare providers should consider the potential effects and proposed mechanisms of IASTM to evaluate the precautions, contraindications, treatment scenario, treatment goals, and IASTM application specifics (e.g., force, duration, etc.) within unique patient encounters to fully evaluate the list of precautions and contraindications identified in this study [1,4,17].

### 4.1. Practice Implications

This modified Delphi survey should be considered an update from past publications discussing IASTM precautions and contraindication. To date, a 2019 IASTM narrative review [1] is the most current publication that proposed 26 precautions, 26 contraindications, and 9 conditions considered as both. The narrative review compiled the list from a 2008 IASTM case series [10], different IASTM manufacturer continuing education courses [6,7,8,9], and safety guidelines from the therapeutic massage and Gua Sha literature [1]. To date, no controlled studies on IASTM have been conducted on treatment precautions and contraindications. The current study identified 39 conditions or treatment considerations versus 61 conditions from the past narrative review [1]. Interestingly, IASTM experts omitted past notable conditions such as but not limited to petechiae, hypertension, osteoporosis, and diabetes. A full list of past conditions can be found in a 2019 narrative review [1], while Table 6 provides a consolidated list of the 39 conditions for further clinical reference.

Our primary findings were that our expert panel agreed that the presence of patient acute systemic infection, allergies to metals, emollients, or latex, and acute inflammatory skin conditions were strong contraindications to IASTM application, as was IASTM application at or near the site of unhealed or unstable bone fracture, thrombophlebitis or osteomyelitis, open skin wounds, skin scrapes or blisters, and insect bites of unexplained origin (Table 6). Strong agreement was also found for mild/moderate skin hypersensitivity and rheumatoid arthritis as precautions. While few research studies have examined these conditions and corresponding IASTM treatment parameters (e.g., treatment location, force or stroke type applied, treatment durations, etc.), our findings identified potential considerations for clinical practice. For example, researchers [17] have identified that some healthcare professionals will use IASTM in the presence of different types of conditions (e.g., fractures) that were identified by our experts as contraindications or precautions. Thus, further research is needed to understand the clinical application of IASTM considering the proposed precautions and contraindications, including patient scenario-specifics, treatment application parameters, and treatment outcomes expected and produced in those situations. For now, healthcare professionals should consider that the survey list of precautions and contraindications is based upon expert consensus and is a starting point to guide IASTM application.

Healthcare professionals are encouraged to evaluate patient risk factors prior to administering IASTM treatment. This will allow the professional to determine if the treatment would be a precaution or contraindication for the patient. For example, if the patient has a precautionary condition, then IASTM treatment parameters (e.g., treatment technique, treatment dosage time) may have to be modified to ensure safe treatment. If the patient has a contraindicative condition, then a safe alternative treatment should be chosen by the professional in order to avoid any adverse events. Best practice for healthcare professionals also includes educating the patient (prior to treatment) regarding the IASTM treatment process, discussing any precaution and contraindication, answering any related questions, and finally obtaining informed patient consent. This will ensure patients’ autonomy and that they are fully informed prior to IASTM treatment. This may be most relevant among patients with high-risk medical conditions or factors that require treatment modification.

This Delphi survey reported expert consensus on a topic which only provides level 4 evidence [16]. Other conditions or considerations may exist that could be precautionary or contraindicative for some patients, and the expertise of the clinician and unique patient scenario should be considered in individual patient care situations. Certain situations may pose little risk to the patient in consideration of a precaution, while other situations (e.g., the potential to mechanically dislodge a thrombosis) would contain much greater risk and would warrant greater consideration by the healthcare professional [11].

### 4.2. Future Research

The current modified Delphi study using the *strength of agreement grading scale* should be considered a positive progression of the current evidence and a starting point for future controlled research. This modified Delphi study was needed to establish contemporary expert consensus on IASTM precautions and contraindications and to gather information for future controlled studies on this topic [1,15]. Thus, the results should serve as an initial step in a series of planned IASTM studies investigating such topics as treatment precautions and contraindications, indications, physiological and therapeutic effects, and pre- and post-treatment outcomes Further, this IASTM modified Delphi survey should be repeated every 5 years to update expert consensus on precautions, contraindications, or both, as well as other considerations (e.g., treatment indications), to allow researchers to analyze changes in expert opinions over time, identify other areas of needed research, and further develop methodologies for controlled studies.

### 4.3. Limitations

Several limitations need to be discussed for this modified Delphi survey study. First, this survey was sent to a group of identified IASTM experts. A larger, more diverse sample of healthcare professionals who use IASTM in clinical practice may have produced different results. However, to the researchers’ knowledge, this is the first study using Delphi methodology to explore expert consensus on IASTM precautions and contraindications. Second, while IASTM experts had 100% agreement for the final list of 39 precautions, contraindications, or both using the *strength of agreement grading scale*, healthcare professionals should consider that the list is not all-inclusive. Other IASTM medical conditions or treatment considerations may exist for different patients that should be considered, and our methodology did not allow for the assessment of risk of IASTM application or how different patient scenarios may alter the risk in relation to applying IASTM when a precaution or contraindication is present. Third, the study results can only be generalized to the healthcare professionals surveyed. Other healthcare professionals or those who work with specialized patient populations may have provided different responses that are more applicable to specific patient populations or professional settings.

## 5. Conclusions

This IASTM modified Delphi study was the first survey to document expert consensus on precautions andcontraindicationsbased upon the strength of agreement. The IASTM medical conditions and considerations documented in this survey should be considered a starting point for healthcare professionals since other conditions may exist beyond this study that may need to be considered for some patients. This survey should be the first step in a series of planned IASTM studies to establish the best-practice recommendations for the application of IASTM in clinical practice.

## Figures and Tables

**Table 1:** Medical conditions and treatment considerations for IASTM precautions and contraindications.

Musculoskeletal Conditions	Cardiovascular, Respiratory, and Chronic Conditions	Integumentary, Connective Tissue, Nervous System, and Psychological Conditions	Miscellaneous Conditions And Treatment Considerations
Acute ligament sprain	Asthma	Abnormal skin sensation (e.g., numbness)	Allergies to metals, emollients, latex
Acute muscle strain and/or tendon injury	Bleeding disorders (hemophilia)	Acute inflammatory skin conditions	Acute systemic infection (viral or bacterial), fever, or contagious condition
Ankylosing spondylitis	Cancer or malignancy	Alzheimer’s disease and dementia	Body art
Bone surgical hardware	Chronic obstructive pulmonary disease (COPD)	Amyotrophic lateral sclerosis (ALS)	Direct pressure over face, eyes, body prominences, arteries, veins, or nerves.
Chronic muscle strain and/or tendon injury	Diabetes	Chronic regional pain syndrome	Flu/flu-like symptoms
Chronic ligament sprain	Heart disease	Connective tissue disorders Epilepsy	Herbal supplements
Delayed onset of muscle soreness	Hypertension (controlled)	Fear avoidance	Inability to position body during treatment
Hematoma	Hypertension (uncontrolled)	Guillain–Barre Syndrome	Inability to communicate (e.g., language barrier, cognitive issues)
Local joint or tissue inflammation	Kidney dysfunction or disease	Healing surgical scars	Insulin pump (e.g., around device)
Lupus	Liver disease	Infection: meningitis, encephalitis	Medications that alter sensation
Myositis ossificans	Lymphedema	Insect bites of unexplained origin	Medications that thin blood
Osteoarthritis	Metabolic syndrome	Local tissue inflammation	Medications such as hormone replacement or fluoroquinolone antibiotics
Osteopenia	Peripheral vascular disease/insufficiency, varicose veins	Mild/moderate skin hypersensitivity	Post injection (e.g., steroid, PRP)
Osteoporosis	Thrombophlebitis or osteomyelitis	Open skin wounds	Pacemaker (e.g., around device)
Polymyositis		Multiple sclerosis	Patient age (e.g., young child)
Psoriatic arthritis		Parkinson’s disease	Pregnancy (without MD consultation)
Rheumatoid arthritis		Petechiae	Pregnancy (high risk)
Severe scoliosis or spinal deformity		Psoriasis	Severe pain felt by patient
Unhealed or unstable bone fracture		Psychological condition that would affect a patient’s response to treatment	Substance abuse (e.g., alcohol, drugs)
Unhealed bone stress fracture		Severe skin hypersensitivity	
		Skin burn scars	

**Table 2:** Strength of agreement grade scale.

Grade	Definition
A (Strong)	>70% expert agreement
B (Moderate)	69–60% expert agreement
C (Weak)	59–50% expert agreement
D(* Both)	<50% expert agreement

* Both: Condition can potentially be a precaution and contraindication.

**Table 3:** IASTM precautions.

Strength of Agreement	Musculoskeletal Conditions	Cardiovascular, Respiratory, and Chronic Conditions	Integumentary, Connective Tissue, Nervous System, and Psychological Conditions	Miscellaneous Conditions and Treatment Considerations
A (Strong) (≥70% agreement)	- Rheumatoid arthritis		- Mild/moderate skin hypersensitivity	
B (Moderate) (69–60% agreement)	- Psoriatic arthritis		- Psychological condition that would affect a patient’s response to treatment	- Flu or flu-like symptoms - Medications that thin blood - Medications such as hormone replacement or fluoroquinolone antibiotics
C (Weak) (59–50% agreement)	- * De Quervain’s tenosynovitis		- Psoriasis - Skin burn scars - Fibromyalgia with nervous system sensitivity - * Abnormal skin sensation (e.g., numbness, tingling)	- Medications that alter sensation - Severe pain felt by patient - Inability to position body during treatment

* Condition received 23/24 responses.

**Table 4:** IASTM contraindications.

Strength of Agreement	Musculoskeletal Conditions	Cardiovascular, Respiratory, and Chronic Conditions	Integumentary, Connective Tissue, Nervous System, and Psychological Conditions	Miscellaneous Conditions and Treatment Considerations
A (Strong) (≥70% agreement)	- Unhealed or unstable bone fracture	- Thrombophlebitis or osteomyelitis	- Acute inflammatory skin conditions - Open skin wounds - Skin scrapes or blisters - Insect bites of unexplained origin	- Allergies to metals, emollients, and latex - Acute systemic infection (viral or bacterial), fever, or contagious condition
B (Moderate) (69–60% agreement)	- Myositis ossificans	- Bleeding disorders (e.g., hemophilia)	- Severe skin hypersensitivity - Skin rash	- Pacemaker (e.g., treatment around device) - Insulin pump (e.g., treatment around device)
C (Weak) (59–50% agreement)	- Polymyositis			- Direct pressure over face, eyes, body prominences, arteries, veins, or nerves

**Table 5:** Both IASTM precautions and contraindications).

Strength of Agreement	Musculoskeletal Conditions	Cardiovascular, Respiratory, and Chronic Conditions	Integumentary, Connective Tissue, Nervous System, and Psychological Conditions	Miscellaneous Conditions and Treatment Considerations
D (Both) (<50% agreement)	- Unhealed bone stress fracture - * Dupuytren’s contracture	- Peripheral vascular disease or insufficiency, varicose veins - Cancer and malignancy - * Lupus	- Healing surgical scars	- Inability to communicate (e.g., language or cognitive issues) - Pregnancy (high risk)

* Condition received 23/24 responses.

**Table 6:** Consolidated list of precautions, contraindications, or both.

Strength of Agreement Grade	Precautions	Contraindications
A (Strong) (≥70% agreement)	- Mild/moderate skin hypersensitivity - Rheumatoid arthritis	- Unhealed or unstable bone fracture - Thrombophlebitis or osteomyelitis - Acute inflammatory skin conditions - Open skin wounds - Skin scrapes or blisters - Insect bites of unexplained origin - Allergies to metals, emollients, and latex - Acute systemic infection (viral or bacterial), fever, or contagious condition
B (Moderate) (69–60% agreement)	- Flu or flu-like symptoms - Medications that thin blood - Medications such as hormone replacement or fluoroquinolone antibiotics - Psoriatic arthritis - Psychological condition that would affect a patient’s response to treatment	- Myositis ossificans - Bleeding disorders (e.g., hemophilia) - Severe skin hypersensitivity - Skin rash - Pacemaker (e.g., treatment around device) - Insulin pump (e.g., treatment around device)
C (Weak) (59–50% agreement)	- Abnormal skin sensation (e.g., numbness, tingling) - De Quervain’s tenosynovitis - Fibromyalgia with nervous system sensitivity - Inability to position body during treatment - Medications that alter sensation - Psoriasis - Skin burn scars - Severe pain felt by patient	- Polymyositis - Direct pressure over face, eyes, body prominences, arteries, veins, or nerves
D (Both *) (<50% agreement)	- Cancer and malignancy - Dupuytren’s contracture - Healing surgical scars - Inability to communicate (e.g., language or cognitive issues) - Lupus - Peripheral vascular disease or insufficiency, varicose veins - Pregnancy (high risk) - Unhealed bone stress fracture	

* Both: Condition can potentially be a precaution and contraindication.

## Data Availability

Data analyzed for this systematic review are from published manuscripts appraised in the manuscript. These studies and data are available on electronic databases or the respective journal websites.

## Supplementary Materials

The following supporting information can be downloaded at: https://www.mdpi.com/article/10.3390/healthcare13060642/s1, IASTM Delphi Expert Panel and Glossary of Terms
