# Supplementary material for: International Expert Consensus on Instrument-Assisted Soft-Tissue Mobilization Precautions and Contraindications: A Modified Delphi Study
Source: Healthcare (Basel). 2025 Mar 15;13(6):642. doi: 10.3390/healthcare13060642 (PMC11941819; doi:10.3390/healthcare13060642)
Supplement: Supplementary file 1 [file healthcare-13-00642-s001.zip › MDPI HC IASTM Supplementary File .pdf]

**Supplementary File**
**IASTM Delphi Expert Panel**

This Delphi study included 24 IASTM experts that participated in all 3 rounds. The results section on the manuscript provides basic respondent demographic details. Further details are provided below:

### Expert Healthcare Education

- Doctor of Physical Therapy: *13 experts*
- Doctor of Chiropractic: *6 experts*
- Doctor of Athletic Training: *4 experts*
- Doctor of Occupational Therapy: *1 expert*
- Other academic degrees (e.g. PhD, DSc,): *6 experts*

### Expert Healthcare Clinical Practice

- Current IASTM clinical practice: *24 experts*

### Published Studies

- IASTM published research: *18 experts*

### Actively teaching IASTM (e.g. professionals, students, etc.)

- Teaching IASTM courses and content: *24 experts*
