# Supplementary material for: International Expert Consensus on Instrument-Assisted Soft-Tissue Mobilization Precautions and Contraindications: A Modified Delphi Study
Source: Healthcare (Basel). 2025 Mar 15;13(6):642. doi: 10.3390/healthcare13060642 (PMC11941819; doi:10.3390/healthcare13060642)
Supplement: Supplementary file 1 [file healthcare-13-00642-s001.zip › MDPI Healthcare Supplementary Glossary.pdf]

MDPI Healthcare: IASTM Delphi IASTM Study

Supplemental Glossary

Medical Condition	Definition
Ankylosing spondylitis	A type of arthritis that causes inflammation and stiffness in certain parts of the spine.
Chronic obstructive pulmonary disease (COPD)	A group of lung diseases that cause ongoing breathing problems.
Chronic regional pain syndrome	A neurological condition that causes pain, changes in skin color and other symptoms in limb or other body part.
Metabolic syndrome	A group of conditions that increase the risk for cardiovascular disease, type 2 diabetes, and stroke.
Myositis ossificans	A condition where bone tissue forms with the muscle.
Myofascial	A term that refers to both muscle and fasciae connective tissue.
Osteomyelitis	A term that refers to a bone infection.
Petechiae	local non-blanching red or purple sports that appear on the skin due to bleeding under the skin surface.
Polymyositis	an inflammatory muscle disease characterized by muscle weakness, pain, and fatigue.
Thrombophlebitis	a condition where a blood clot (or thrombus) forms in a vein and causes inflammation.
